# Characterization of the complete chloroplast genome of *Betula chichibuensis* (Betulaceae), a critically endangered limestone birch

**DOI:** 10.1080/23802359.2020.1768932

**Published:** 2020-05-27

**Authors:** Takuya Yoshida, Yuji Igarashi, Toshihide Hirao

**Affiliations:** a The University of Tokyo Forests, Graduate School of Agricultural and Life Sciences, The University of Tokyo, Tokyo, Japan; b The University of Tokyo Chichibu Forest, Graduate School of Agricultural and Life Sciences, The University of Tokyo, Chichibu, Japan

**Keywords:** *Betula chichibuensis*, Betulaceae, chloroplast genome, conservation, critically endangered species

## Abstract

*Betula chichibuensis* is a critically endangered limestone birch confined to the Chichibu and Kitakami mountains in central and northeastern Japan, respectively. In this study, we assembled and characterized the complete chloroplast genome of *B. chichibuensis*. The whole chloroplast genome was 160,791 bp in length, consisting of a large single-copy (LSC) region of 89,504 bp and a small single-copy (SSC) region of 19,175 bp, separated by a pair of inverted repeat (IR) regions of 26,056 bp. It contained 133 genes, including 88 protein-coding genes (80 PCG types), 37 tRNA genes (30 tRNA types), and eight rRNA genes (four rRNA types). The overall GC content of the chloroplast genome was 36.01%. Phylogenetic analysis resolved *B. chichibuensis* as sister to the clade containing *B. pendula*.

The genus *Betula* L. (Betulaceae) consists of approximately 60 tree species mainly distributed in the Northern Hemisphere (Furlow 1990). *Betula chichibuensis* H. Hara (subgenus *Aspera*) is a small tree that grows only on limestone outcrops in the mountains (Ashburner and McAllister 2013). *Betula chichibuensis* is significantly distinct from other birches because it has ovate eglandular leaves with up to 18 pairs of lateral veins and with long hairs along the midrib and lateral veins, and clusters of numerous male catkins from several buds toward the ends of the twigs (McAllister 2019). This species is endemic to Japan and assessed as Critically Endangered in the IUCN Red List (Shaw et al. 2014). It is confined to the Chichibu and Kitakami mountains in central and northeastern Japan, respectively (Igarashi et al. 2017). In this study, we first report the complete chloroplast genome of *B. chichibuensis*, which will offer a useful resource for future conservation genetics.

Fresh leaves of *B. chichibuensis* were collected from a single individual along the Oku-Chichibu Forest Road (35°57´N, 138°44´E) in the Chichibu Mountains of Japan. The voucher specimen was deposited at the Herbarium of the University of Tokyo Chichibu Forest (voucher number UTCFBC00085). The total genomic DNA was extracted using the DNeasy Plant Mini Kit (QIAGEN GmbH, Hilden, Germany) and sequencing was performed on an Illumina MiSeq platform (Illumina Inc., San Diego, CA, USA). The filtered reads were de novo assembled with NOVOPlasty (Dierckxsens et al. 2017). The assembled chloroplast genome was annotated using the online annotation tool GeSeq (Tillich et al. 2017). The annotation was checked and corrected by referring to the chloroplast genomes of related species, *B. nana* (KX703002) and *B. costata* (MN830400), using Geneious Prime 2020.0.3 (Biomatters Ltd., Auckland, New Zealand).

The complete chloroplast genome of *B. chichibuensis* (GenBank Accession No. LC542973) was 160,791 bp in length and had a typical quadripartite structure, consisting of a large single-copy (LSC) region of 89,504 bp and a small single-copy (SSC) region of 19,175 bp, separated by a pair of inverted repeat (IR) regions of 26,056 bp. It contained 133 genes, including 88 protein-coding genes (80 PCG types), 37 tRNA genes (30 tRNA types), and eight rRNA genes (four rRNA types). Most of the genes were located in the single-copy region, while 19 genes were duplicated in the IR regions. The overall GC content of the chloroplast genome was 36.01% while that of the LSC region, SSC region, and IRs was 33.64%, 29.50%, and 42.48%, respectively.

To determine the phylogenetic position of *B. chichibuensis*, a phylogenetic tree was constructed using the complete chloroplast genomes of *B. chichibuensis*, 10 *Betula* species and one *Alnus* species as the outgroup. All of the chloroplast genomes were aligned with MAFFT version 7 (Katoh et al. 2019), and maximum-likelihood phylogenetic inference was performed under the GTR + G + I model using RAxML-NG (Kozlov et al. 2019). Bootstrap values were calculated with 1000 replicates. The phylogenetic tree showed *B. chichibuensis* as sister to the clade containing *B. pendula* (Figure 1). This result provides new insight for phylogenetic studies of *Betula* and further conservation strategies for *B. chichibuensis*.

**Figure 1.**
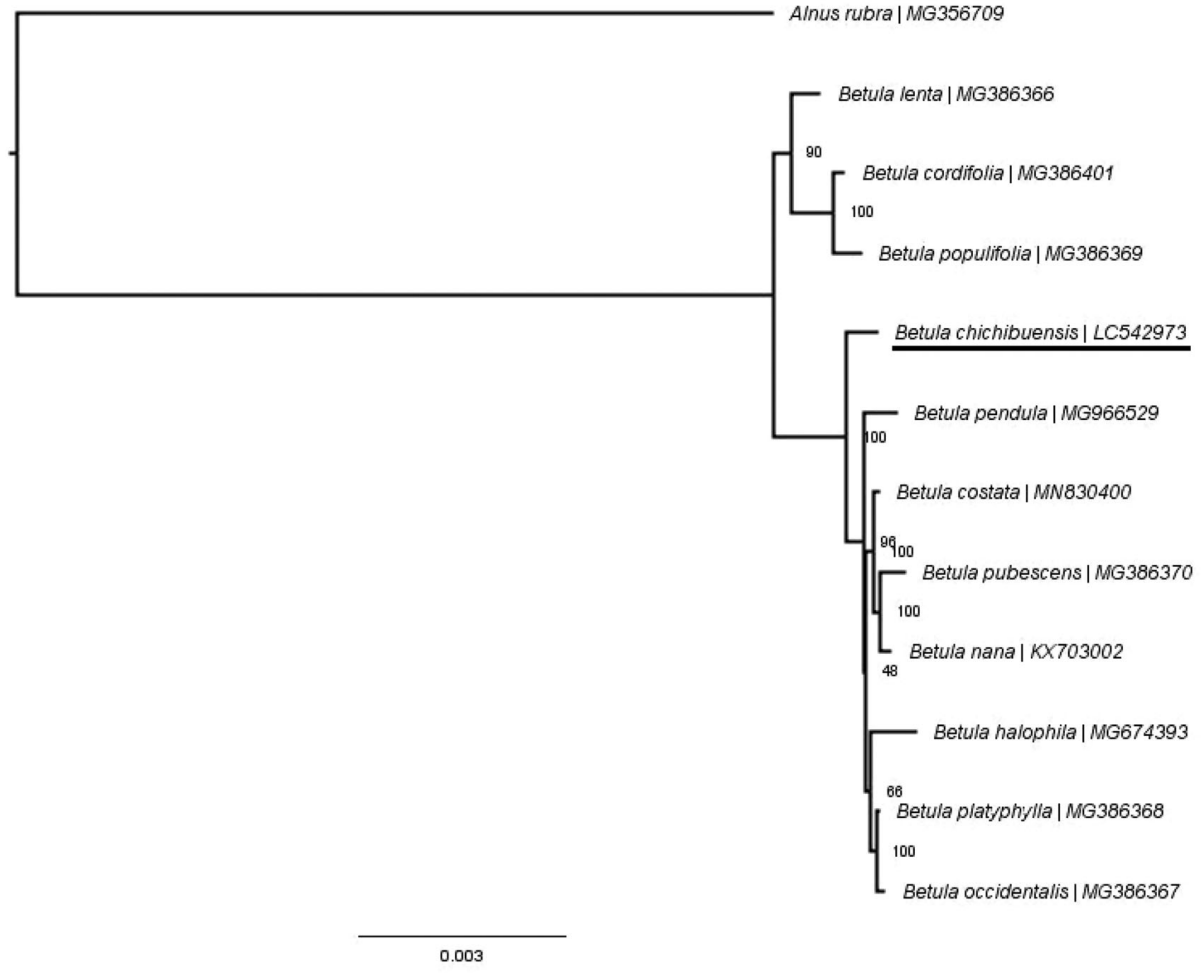
Maximum-likelihood phylogenetic tree based on the complete chloroplast genomes of 11 *Betula* and one *Alnus* species as the outgroup. Bootstrap values are shown on each node.

## Data Availability

The data that support the findings of this study are openly available in the National Center for Biotechnology Information (NCBI) at https://www.ncbi.nlm.nih.gov/nuccore/LC542973.1, with the accession number LC542973.1.
